# Group Lasso Regularized Deep Learning for Cancer Prognosis from Multi-Omics and Clinical Features

**DOI:** 10.3390/genes10030240

**Published:** 2019-03-21

**Authors:** Gangcai Xie, Chengliang Dong, Yinfei Kong, Jiang F. Zhong, Mingyao Li, Kai Wang

**Affiliations:** 1 Center for Cellular and Molecular Therapeutics, Children’s Hospital of Philadelphia, Philadelphia, PA 19104, USA; gcxiester@gmail.com (G.X.); coco90417@gmail.com (C.D.); 2 Mihaylo College of Business and Economics, California State University Fullerton, Fullerton, CA 92831, USA; yikong@fullerton.edu; 3 Division of Periodontology, Diagnostic Sciences and Dental Hygiene, and Division of Biomedical Sciences, Herman Ostrow School of Dentistry, University of Southern California, Los Angeles, CA 90089, USA; jzhong@usc.edu; 4 Department of Biostatistics, Epidemiology & Informatics, University of Pennsylvania Perelman School of Medicine, Philadelphia, PA 19104, USA; mingyao@pennmedicine.upenn.edu; 5 Department of Pathology and Laboratory Medicine, University of Pennsylvania Perelman School of Medicine, Philadelphia, PA 19104, USA

**Keywords:** deep learning, genomics, cancer, survival analysis

## Abstract

Accurate prognosis of patients with cancer is important for the stratification of patients, the optimization of treatment strategies, and the design of clinical trials. Both clinical features and molecular data can be used for this purpose, for instance, to predict the survival of patients censored at specific time points. Multi-omics data, including genome-wide gene expression, methylation, protein expression, copy number alteration, and somatic mutation data, are becoming increasingly common in cancer studies. To harness the rich information in multi-omics data, we developed GDP (Group lass regularized Deep learning for cancer Prognosis), a computational tool for survival prediction using both clinical and multi-omics data. GDP integrated a deep learning framework and Cox proportional hazard model (CPH) together, and applied group lasso regularization to incorporate gene-level group prior knowledge into the model training process. We evaluated its performance in both simulated and real data from The Cancer Genome Atlas (TCGA) project. In simulated data, our results supported the importance of group prior information in the regularization of the model. Compared to the standard lasso regularization, we showed that group lasso achieved higher prediction accuracy when the group prior knowledge was provided. We also found that GDP performed better than CPH for complex survival data. Furthermore, analysis on real data demonstrated that GDP performed favorably against other methods in several cancers with large-scale omics data sets, such as glioblastoma multiforme, kidney renal clear cell carcinoma, and bladder urothelial carcinoma. In summary, we demonstrated that GDP is a powerful tool for prognosis of patients with cancer, especially when large-scale molecular features are available.

## 1. Introduction

Survival analysis, which models time-to-event outcomes, has been widely adopted in cancer studies, for example the docetaxel chemotherapy study for prostate cancer [1], pertuzumab effects on breast cancer therapies [2], and immunoscore on colorectal cancer patient survival [3]. One important feature of survival analysis is that part of the observed data is censored, in which the expected event did not happen to the cancer patients at the end of the study or the patients were not followed up on [4]. In order to study the effects of different covariates on partially censored survival time, a time constant hazard rate model named Cox proportional hazard model (CPH), proposed by David R. Cox decades ago [5], has been widely used in clinical research.

With the advent of high-throughput sequencing technologies [6], various genome-wide covariates of survival time, such as gene expression, DNA mutation, and copy number variation (CNV), has been profiled for the cancer patients. Notably, recent progress in international cancer projects, for example The Cancer Genome Atlas (TCGA) project [7], provides researchers ample opportunities to freely access genome-wide cancer data. At a recent data portal release of TCGA, there were more than 3 million mutations and over 20 thousand genes that were profiled. However, compared to the high dimensionalities of molecular features, the number of the cancer patients is usually very small. For example, in one of the most recent TCGA study about the genomic studies of adult soft tissue sarcomas [8], there were only 206 samples that were available for analysis.

The small sample size raised questions about the application of the Cox model to the survival analysis of cancer patients using molecular features with high dimensionalities. When the number of features or covariates is significantly larger than the number of patients studied, it has been suggested that traditional the Cox model cannot been directly applied [9]. However, shrinkage-based methods or regularization methods have been proposed to be useful to deal with high dimension, low sample size data in survival analysis [9]. One of the regularization methods is the lasso proposed by Robert Tibshirani for linear regression models [10]. Lasso stands for “least absolute shrinkage and selection operator,” and it can retain the coefficients of good features by shrinking the coefficients of other features to be 0. Lasso integrated with the Cox model showed the ability to shrink part of the coefficients of the Cox model to be 0 [11]. Furthermore, other regularization methods, such as elastic net [12], have also been shown to be powerful for variable selection.

In cancer studies, various molecular features might contribute coordinately to disease initiation, progression, and prognosis [13]. For example, the mutations in cancer driver genes can also lead to the changes of gene expression and protein expression. As one of the significant extensions of the lasso method, group lasso had been proposed to be able to use group information to reduce the dimensionality of the input data and performs better in the data with prior group knowledge [14]. A study of group lasso for logistic regression [15] found that a group lasso could help to give consistent prediction for the data with high dimensionality of features with a small sample size. In the genome-wide cancer studies, the number of molecular features is usually much larger than the number of patients; however, only a few of them might contribute to the disease prognosis. For those cancer studies, group lasso might be helpful to select the most relevant molecular features and to reduce the dimensionality of those features.

Besides regularization methods, deep learning, which is a machine learning method with significant progresses in recent years, has been illustrated to be an efficient machine learning method for revealing new discoveries from high-dimensional data [16]. It has been successfully used in playing the game of go [17], generating natural language descriptions for images [18], and applied to autonomous driving [19]. Not only being widely adopted in those areas, it has also been applied to biological studies, for instance, the prediction of transcription factor binding sites [20], splicing code prediction [21], and calculating non-coding variants effects [22].

Furthermore, deep learning has also been applied to the survival studies in recent years. DeepSurv, a deep learning based framework combined with cox model, performed well with cancer data with high-dimensional covariates [23]. SurvivalNet, combined with Bayesian optimization methods, had been applied to high-dimensional survival predictions in cancer [24]. However, those studies did not consider the group prior knowledge in the molecular features for the survival analysis in cancer.

In this study, we proposed a new integrated method and provided an open-source python package named GDP (Group lass regularized Deep learning for cancer Prognosis) for cancer survival analysis by taking advantage of the gene-level group prior knowledge. The GDP integrated group lasso regularization method, tensorflow [25] based deep learning framework, and CPH model were used to analyze partially censored cancer survival data. It shows higher accuracy compared to the lasso method for the input with group prior knowledge in both simulated and real cancer survival data.

## 2. Materials and Methods

### 2.1. Data Collection

In general, the data used in this study contained two types of datasets, one was the simulated data and another was the TCGA cancer data. The generation of the first dataset is described in the method part below, and the TCGA datasets were downloaded from Broad GDAC (Genome Data Analysis Center) Firehose (https://gdac.broadinstitute.org/) and TCGA data portal (https://cancergenome.nih.gov/).

In detail, fbget provided by Broad Institute (https://confluence.broadinstitute.org/display/GDAC/fbget) was used to download RSEM [26] (RNA-seq by expectation-maximization) normalized gene expression data, GISTIC2.0 (Genomic Identification of Significant Targets in Cancer) [27] processed copy number variation (CNV), normalized RPPA protein expression data, Variant Call Format [28] (VCF) cancer DNA somatic mutation data, and part of the clinical data from Broad GDAC Firehose. Furthermore, the remaining datasets that were not found in GDAC were downloaded from the TCGA data portal.

### 2.2. TCGA Data Preprocessing

The whole data preprocessing procedure contained four steps, data cleaning, data imputation, data transformation, and data normalization. At the data cleaning step, the features with 80% or more missing values were discarded, and at the data imputation step, the mean value was used if the feature values in some patients were missing. Furthermore, during the data transformation step, categorical data was converted to numerical data, and all the molecular value was normalized into standard score (z score). For DNA mutation data, iCAGES (Integrated CAncer Genome Score) [29] was applied to process the data, and the ICAGES gene scores were used. The ICAGES gene scores were calculated from the logistic regression model at gene prioritization stage in ICAGES. For DNA CNV data, GISTIC tool [27] was used to process the data, and the focal data by gene output was used in this study. The TCGA tumor types used in this study can be found in Table S1.

### 2.3. Data Simulation

As suggested by previous study [30], the latent survival time was calculated according to following formula:

(1) $$T_{l}={\left(-\frac{\log\left(U\right)}{\lambda \exp\left(X\beta \right)}\right)}^{1/v}$$

(2) $$X=\left[\;X_{1},\;X_{2},\ldots ,X_{G}\right]$$

(3) $$\beta =\left[\beta_{1},\beta_{2},\ldots ,\beta_{G}\right]$$

where *λ* (8 × 10^−7^) is the scale parameter and v (2.8) is the shape parameter of Weibull distribution. $X_{i}$ (i = 1, 2, …, G) is the feature vector with the length of the group size, and it is generated from multivariate normal distribution (covariance matrix with diagonal elements set to be 1 and other elements set to be 0.3). G is the number of groups. *U* is randomly generated from a uniform distribution with the interval between 0 and 1. *β_i_* is the coefficients vector for group *i* with the length of group size, and the value of it depends on the type of group. If group *i* is the relevant survival time (this group is called relevant group), then each element of *β_i_* is drawn from the normal distribution with mean of 0 and standard deviation of $\frac{10}{\#\;of\;relevant\;features}$; otherwise *β_i_* is set to be a vector of all zeros.

The censoring time $T_{c}$ was randomly drawn from the exponential distribution with the probability density function of:

(4) $$f\left(x\right)=\lambda_{c}\exp\left(-\lambda_{c}x\right),\;x\geq 0$$

where $\lambda_{c}$ (5 × 10^−3^) is the rate of exponential distribution. Furthermore, the event status (*S* or survival status) and censoring status (C) were formulated according to:

(5) $$S=\{\begin{array}{c} 1\;if\;T_{c}\geq T_{l}\\ 0\;if\;T_{c}<T_{l} \end{array}$$

(6) C=1−S

Furthermore, the observed time ($T_{o}$) was simulated according to:

(7) $$T_{o}=\;\min\;\left(T_{c},T_{l}\right)$$

In the comparison between GDP and CPH, because the simulation was based on CPH, there was a bias for CPH. In order to overcome this bias, we added another layer of function *s* to the latent survival time simulation model.

(8) $$T_{l}={\left(-\frac{\log\left(U\right)}{\lambda \exp\left(s\left(X\right)\beta \right)}\right)}^{1/v}$$

*S* is either identical function (no transformation of the feature matrix) or a non-linear function (transformation of the feature matrix by either a quadratic function or absolute function).

Although this simulation is a simplified reflection of the real TCGA data, certain simulation parameters were set according to the average data size of the selected TCGA tumor samples, including the group size, feature size, and sample size. Furthermore, the detailed information of the simulation settings can be found in Table S2. Furthermore, the R code used for the simulation can be found in our GitHub GDP repository (https://github.com/WGLab/GDP/tree/master/simulation).

### 2.4. GDP Model

**Figure 1:**
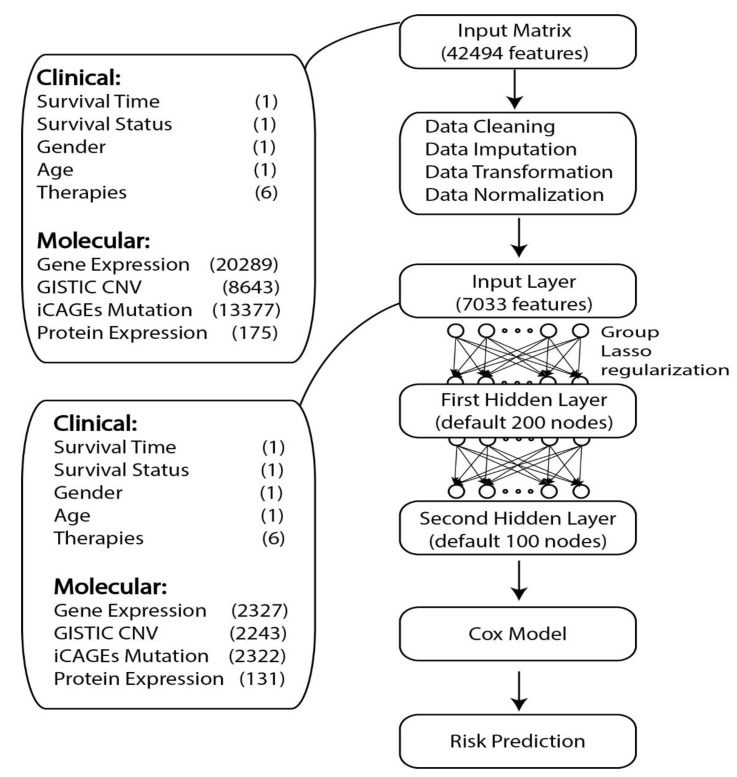
Basic framework of GDP. GDP can perform survival analysis for the cancer data with high-dimensional features. The number of features for each type of datasets is given in the parenthesis. GISTIC CNV means the CNV data was processed using GISTIC 2.0 and the focal data via gene output was used. Furthermore, iCAGES mutation means the DNA mutation data was processed using iCAGES and the iCAGES gene level scores were used. The number of molecular features shown here is the average number of molecular features from 14 TCGA tumor types selected in this study.

The whole GDP model contained three components: the first one was a fully connected deep learning framework with two hidden-layers, the second one was the CPH module connected to the output of the first part, and the third one was the group lasso regularization method applied to regularize the coefficients of the input layer of the neural network. Group prior knowledge used in this study meant different features from the same gene could be grouped together during regularization such as the copy number variation information, gene expression level, protein expression level, and single nucleotide polymorphism of one gene. The detailed information about GDP framework can be found in Figure 1.

Set *X* (features matrix) to be the input layer matrix of GDP neuron network, and the first component of the GDP is:

(9) $$H_{1}=g\left(W_{1}X+B_{1}\right)$$

(10) $$H_{2}=g\left(W_{2}H_{1}+B_{2}\right)$$

*H*_1_ is the first hidden layer of the GDP and *H*_2_ is the second hidden layer, where *g* is the activation function of the neural network, where ReLU (rectified linear unit) was specifically used with the definition:

(11) f(x)=max(0,x)

The hazard module follows the CPH model but using the output of deep learning as the input, and the corresponding formula is:

(12) $$\lambda \left(t|X\right)=\lambda_{0}\left(t\right)\exp\left(\beta^{T}H_{2}\right)$$

where $\lambda_{0}\left(t\right)$ is the baseline hazard at event time *t*, which can be estimated empirically using a standard survival analysis estimator.

Similar to the CPH model, the partial log-likelihood of the GDP hazard model is:

(13) $$l\left(\beta \right)=\underset{i:S_{i}=1}{\sum }\left(\beta^{T}H_{2i}-\log\left(\underset{j:T_{oj}\geq T_{oi}}{\sum }\exp\left(\beta^{T}H_{2j}\right)\right)\right)$$

The optimization of GDP parameters is done through minimizing the cost function, which is defined as follows:

(14) $$C=-l\left(\beta \right)+scale\times \left(alpha\times \overset{L}{\underset{l=1}{\sum }}\sqrt{p_{l}\|W_{1l}\|{}_{2}}+\left(1-alpha\right)\times \|W_{o}\|{}_{1}\right)$$

where $p_{l}$ is the group size for the lth group, and the group lasso was applied to the parameters between input layer and the first hidden layer ($W_{1}$). Furthermore, $W_{o}$ is the other parameter in the model that needs to be trained, α and scale are the parameters for the regularization terms. α controls the proportion of group lasso regularization value and scale regulates the proportion of the whole regularization value. ${\left\|W\right\|}_{p}$ is the p-norm of the weights vector *W*.

### 2.5. Model Training

During each GDP training cycle, one batch (default batch size 50) of the training data was fed into the neural network and the parameters of GDP were optimized through gradient descent. Each batch of the training data was generated by randomly fetching subsets of data from the total training data. Such a cycle of training process was defined as one training step. Feeding GDP with one batch of training data rather than the whole datasets could make it more adaptable to the datasets with bigger sample sizes.

### 2.6. Model Evaluation and Feature Selection

Concordance index (c-index) is commonly used in survival analysis to evaluate the goodness of the model. C-index is the ranked correlation coefficients that measures the proportion of the correctly ranked pairs among all the comparable pairs according to the survival time of patients [31]. Specifically, the c-index is calculated according to:

(15) $$c\text{-}\mathrm{index}=\;\frac{\sum_{\left(i,j\right)\in U}I_{ij}}{Q}$$

where *U* is the union of all comparable pairs, and *Q* is the size of *U*. If the GDP predicted ranking order is the same as the order according to the observed time, then $I_{ij}$ is 1, otherwise it is 0.

During the model evaluation process, GDP was first trained on a training dataset (60% of the patients or simulated samples), and then evaluated on a validation dataset (another 20% of the patients or simulated samples) to select hyper-parameters, and the final model was evaluated on a testing dataset (remaining 20% of the patients or simulated samples). In order to study the training processes, after each training step, the evaluation of the model was done on all three datasets (training, validation, and testing datasets).

After the model was evaluated, the weights from the first layer of final GDP model could be used to select the features that were most relevant to the outcome. First, the absolute value of the weights from the first layer were extracted, and then the sum of the nodes in the first layer for each feature was calculated. Next, for each group, the maximal weights of the features in this group were used for feature selection. Finally, the group-level features were ranked according to the weights. In our study, the molecular features from the same genes were grouped together, and the genes could be ranked according to their weights.

### 2.7. Availabilities of Software

All the statistical analysis was done using homemade R scripts, and the GDP framework was coded in Python language. Both of the R code for simulation and the GDP python sources can be downloaded from the GitHub (https://github.com/WGLab/GDP). GDP was developed on the basis of Google TensorFlow framework, which can be downloaded from https://www.tensorflow.org/.

## 3. Results

### 3.1. Group Lasso Prevents Overfitting During GDP Training

**Figure 2:**
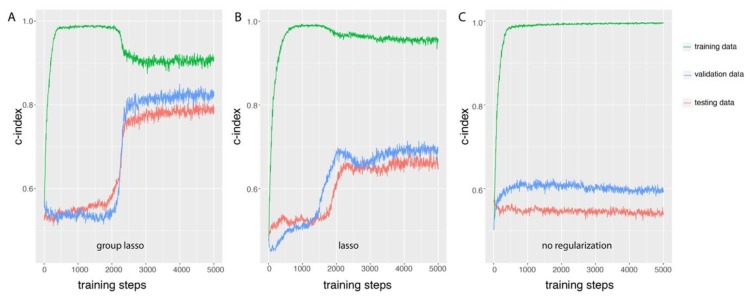
Group lasso overcame the overfitting of GDP training for simulated data with group information as seen in the GDP training process comparison among three different types of regularization methods: (**A**) group lasso, (**B**) lasso, and (**C**) no regularization. One training step is one round of GDP training with 50 randomized samples fed in batch as input, and the model trained after each step was evaluated on training, validation, and testing data. The biggest overfitting gap between testing data and training data was observed in the training process without regularization. Both lasso and group lasso reduced the overfitting gap, and the latter of which more significantly improved the survival prediction accuracy in the testing data (c-index approaches 0.8). For simulation details, see simulation s1 in Table S2.

We first studied the properties of GDP training based on simulated data with group prior knowledge, and the basic framework was the same as the one indicated in Figure 1 except that the input layer data was simulated. The number of features in this simulation was 8000, and was divided into 2000 groups, where 5 groups were set to be directly related to the survival date and status (check s1 in Table S2 for detail information). The c-index evaluation of the models was compared among training, validation, and testing data after each step of training (Figure 2). When regularization methods were applied to GDP (either group lasso Figure 2A or lasso Figure 2B), the change patterns of c-index could be divided into four stages. The first stage was the exponential growth stage, it happens before training steps 500 (group lasso) or 750 (lasso). At this stage, the c-index increased exponentially from random the prediction level of 0.5 to close to perfect prediction level 1 based on the training data. However, the gap between the c-index based on training data and validation data became larger after each round of training. This difference between the prediction accuracies of the GDP model on training data and validation data indicated overfitting of the model for the training data at this stage. The next stage was the stationary stage (500–2000 steps for group lasso and 750–1500 for lasso), where the increasing of the training steps could not further improve the c-index for the training dataset. Interestingly, at the third stage (training steps 2000 to 2500 for group lasso and 1500 to 2000 for lasso), we observed a quick decreasing of the c-index in training data, but a more dramatic increase of the c-index in validation data. The gap of the prediction powers of the GDP model on training data and validation data narrowed at the end of this stage, which illustrated the effects of the regularization method on the GDP training. At this stage, group lasso significantly improved the performance of GDP model on the validation data, from 0.5 to almost 0.8, and lasso also significantly improved the c-index from 0.5 to 0.7. At the final stage, the training process gradually stabilized. As shown in Figure 2A, the survival prediction accuracy of GDP model on testing data was similar to the validation data, and at the stable stage, the c-index was close to 0.8. However, if no regularization method was applied (Figure 2C), the survival prediction performance was much poorer compared to using either group lasso (Figure 2A) or lasso (Figure 2B). If we compare group lasso to lasso, we can see that although the c-index on training data was lower for the group lasso, the improvement of c-index in both of validation and testing data was higher than lasso. Those results illustrate that GDP with regularization methods, especially with group regularization for simulated data with group prior knowledge, could efficiently prevent the model overfitting.

### 3.2. Group Lasso Performed Better than Lasso Regularization on the Simulated Time-To-Event Data with Group Prior Knowledge

We next compared the group lasso regularization method to lasso method on the same simulation datasets used above. In this comparison, we first trained the model with different scenarios of regularization on different GDP scale and α settings. The scale controlled the proportion of the regularization value to the un-regularized loss value, and α controlled the proportion of group lasso regularization.

**Figure 3:**
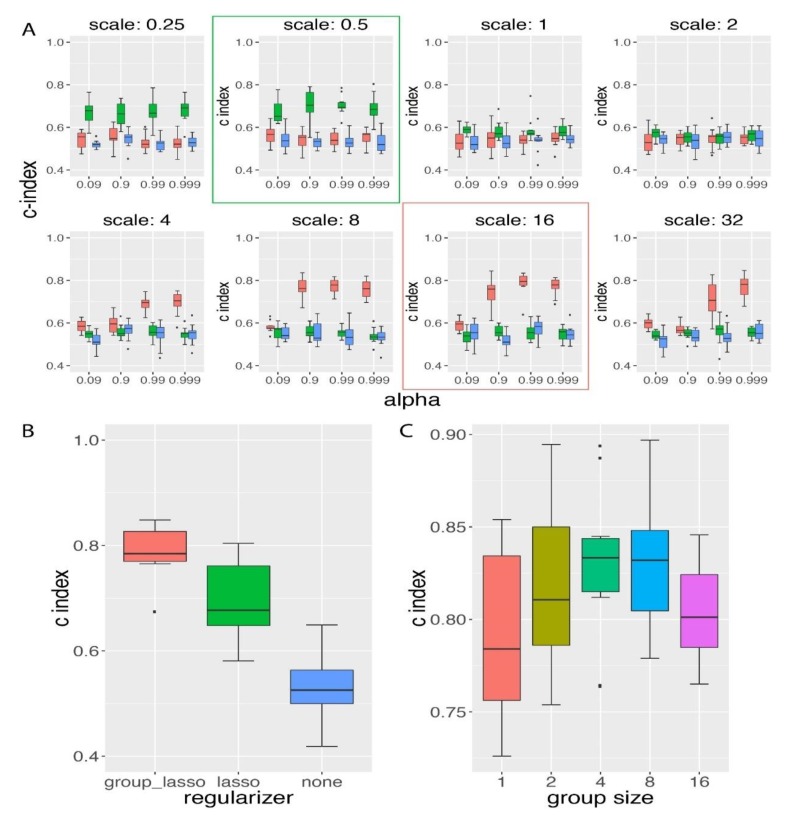
Group lasso regularization method achieved higher survival prediction accuracy than the lasso regularization method. (**A**) Grid search of the best hyper-parameters evaluated on validation data. GDP with group lasso regularization (red) was compared to both of GDP with lasso regularization (green) and no regularization (blue). Group lasso performed best at a scale of 16 (red box), while lasso performed best at a scale of 0.5 (green box). (**B**) Group lasso performed significantly better than both of lasso and no regularization on testing data. The *p*-values based on a two-sided *t*-test between different methods were: 0.0078 (group lasso vs lasso), 1.48 × 10^−8^ (group lasso vs no regularization), and 7.34 × 10^−5^ (lasso vs no regularization). α and scale were the parameters for the regularization terms. α controlled the proportion of group lasso regularization value and scale regulated the proportion of the whole regularization value in the loss function. For simulation details, see s1 in Table S2. (**C**) GDP performance comparison for the simulated data with different group sizes under constant number of relevant features. Simulation settings can be found in s2A–E in Table S2. GDP performed best when the group size was 4 or 8, and performed worst when the group size was 1 (reduced to lasso at group size 1).

As shown by Figure 3A, we had chosen eight scale values and four α values, and lasso achieved the best performance on the validation data at scale of 0.5, while group lasso achieved its best performance on a scale of 16 and an α of 0.99. If we increased the scale level from their best performance scale value, we did not observe further improvement in c-index prediction. In the validation data, group lasso could achieve a c-index value larger than 0.8 on average, and lasso achieved a c-index close to 0.7. In the testing data, we confirmed our observation that group lasso performed significantly better regarding survival hazard modeling than the lasso regularization method (two-sided *t*-test *p*-value of 0.0078; see Figure 3B). At last, we further compared both regularization methods to the scenario of no regularization method applied, and we found that without regularization, GDP performed significantly poorer (Figure 3B). This was consistent with the observation on the high-dimensional data survival analysis in the CPH model [9], where the normal Cox model could not be directly applied to the time-to-event survival data when the number of features was much higher than the number of samples (in this simulation the number of features was 16 times larger than the number of samples).

In conclusion, from the comparative analysis between group lasso and lasso (Figure 3A,B), we found that at the time-to-event survival simulation data with group prior knowledge, group lasso performed significantly better than the lasso regularization method.

### 3.3. Influence of Group Size on the Performance of GDP Survival Prediction

In order to study the influence of group size on the performance of GDP, we compared the GDP performances on the simulation datasets with different group sizes but with a constant number of relevant features (s2A-E in Table S2). The group size ranged from 1 to 16, and the total number of relevant features was kept at 16. When the group size was 1, it has been pointed out by Yuan et al. [14] that the effects of group lasso will be reduced to be similar as lasso. Indeed, we found that when the group size was 1, the average c-index was the lowest among all group sizes (Figure 3C), and when the group size was either of 4 or 8, the average c-index was significantly higher than the one from group size 1 (one-sided *t*-test *p*-value < 0.05). Based on this result, we conclude that under a constant number of relevant features, GDP performed better for the group size that was neither too small nor too large.

### 3.4. GDP Performed Better than CPH under Complex Simulations

**Figure 4:**
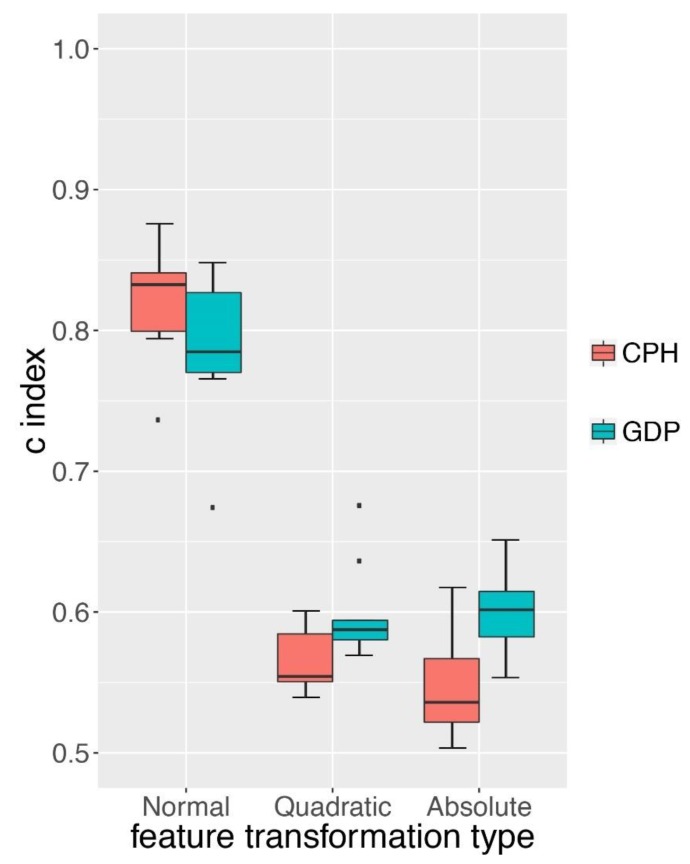
Comparison between GDP and CPH models. C-index comparison between GDP and CPH under different types of feature transformations. Normal: no additional layer of function was applied to the simulation model. Quadratic: quadratic function layer was added to the simulation model. Absolute: absolute function layer was added to the simulation model. Simulation details can be found in S3A–C of Table S2.

The Cox proportional hazard model (CPH) proposed by David Cox has been widely adopted to predict survival hazard [5]. We compared the survival hazard prediction of GDP to CPH on two types of simulation datasets. One was the normal survival simulation data based on the CPH model itself and the other one was based on more complex survival simulation data with one more layer of a non-linear function added to CPH model. We first compared the performance of GDP and CPH on normal simulation data (detail in s3A of Table S2). Although our survival simulation was based on the CPH model, we still observed that GDP had a similar performance to the CPH on this dataset (Figure 4, *p*-value > 0.05). We next examined whether GDP could better handle a more complex survival simulation data by adding an additional layer of a non-linear function to the simulation model (either quadratic function or absolute function). We found that under both scenarios, GDP performed significantly better than CPH (*p*-value < 0.05).

As pointed out by Yann LeCun et al. [16], with enough layers of non-linear transformation within a deep learning network, it could learn very complex functions. The better performance of GDP on quadratic and absolute function transformed simulation data indicated that GDP might be more adapted to complex survival data than the traditional CPH model.

### 3.5. GDP Performances on TCGA Cancer Data

**Figure 5:**
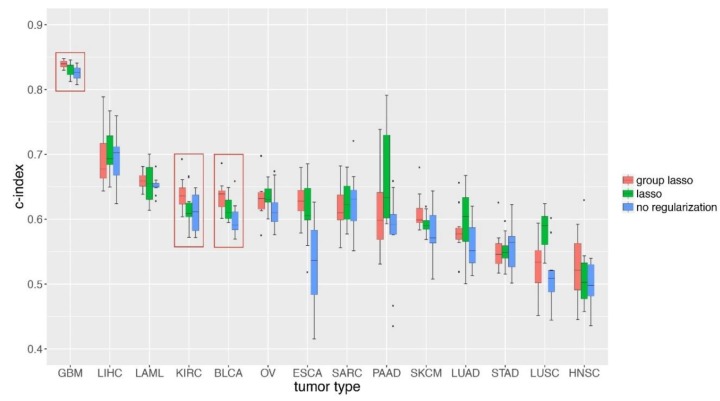
Group lasso performed significantly better regarding survival prediction for GBM, KIRC, and BLCA than the lasso method. GDP survival analysis was done on 14 tumor types from TCGA (Table 1). The group lasso method was compared to lasso and no regularization scenarios. For each tumor type datasets, 20% of the data was kept as testing datasets, and 80% of them was used for training and evaluation. Among this 80%, 75% was used for training and 25% was used for cross-evaluation.

At last, we applied GDP to the TCGA cancer survival data, which contained both of clinical and molecular datasets (Figure 1). We first selected cancer types with both a larger sample size and bigger proportion of uncensored survival time. As shown in Table 1, 14 types of TCGA cancers were selected, and each one contained no less than 100 patients and had a ratio of un-censored no less than 30%. We can see that GBM (glioblastoma multiforme) was ranked first either by sample size or the ratio of uncensored patients. We trained GDP model separately on each cancer type and applied the same grid searching strategies as we did in the simulation studies, then compared the c-index predictions based on different cancer types. As indicated by Figure 5, GDP performed best on GBM data, and the c-index of its performance on GBM testing data was higher than 0.8. The best performance of GDP on GBM data could be explained by its largest sample size and highest ratio of uncensored patients. However, we also observed that the performance of GDP on LIHC (liver hepatocellular carcinoma) was the second best, although neither its sample size nor the ratio of uncensored patients was higher compared to the top ones.

We then compared the performance of GDP based on group lasso to lasso and no regularization methods and found that the group lasso performed significantly better than the other two for GBM, KIRC, and BLCA (Figure 5, Table S1). These results indicate that GDP can be applied to survival analysis of real cancer patients data; however, the number of available individual samples, the proportion of un-censored patients, and the underlying number of genes that are related to the survival of cancer patients should be considered in advance.

Finally, we specifically studied the features that were selected by GDP for GBM. As shown in Table S3, *ING1* was ranked first for predicting GBM survival. It has been reported that the *ING1* gene is a tumor suppressor gene, and it might facilitate tumorigenesis [32]. Additionally, it has also been shown that *ING1* is associated with glioblastoma cells through p53, and that the down-regulation of *ING1* might promote the tumor growth and progression in malignant gliomas [33].

## 4. Discussions

In this study, we developed GDP for the analysis of a cancer prognosis based on both of clinical and high-dimensional molecular features. GDP is the first method to integrate group lasso regularization, deep learning framework, and the Cox model for survival prediction. An important feature of GDP is its ability to take advantage of the group prior knowledge. We also validated its usability in simulated data, showing that with group prior knowledge, GDP could achieve a significantly higher c-index than lasso and a naïve method without any regularization. We further showed that for TCGA data, GDP could improve the prediction accuracy in certain tumor types, such as GBM, BLCA, and KIRC.

The observation in the other 11 tumor types was that adding gene level group information through group lasso method did not significantly improve the prediction ability of the model, which could be due to the heterogeneity of different tumors in terms of the number of causal genes contributing to the survival of the patients, limited sample size, and different ratios of uncensored patients. One limitation of our study is that for the clinical features, we did not have any group prior knowledge to be used by group lasso, and it has been shown that clinical information might play a bigger role in cancer survival predictions than molecular information [34].

One assumption of group lasso is that there is no sparsity within the group, and the method only needs to select features at the group level rather than within group members. The group lasso regularization can be treated as an intermediate between the lasso (l1) and ridge (l2) regularization; when there is only one group, it will be similar to the ridge regularization [14]. One of the properties of ridge regularization is that it will shrink the coefficients to be small enough but most likely not to be zero, and it does not encourage sparsity [14]. Such properties of within-group l2 regularization and between-group l1 regularization makes group lasso inefficient for selecting features within-group. However, if we consider the gene sets belonging to different functional pathways, it is normal that only part of the genes in the relevant pathway are mutated or have their expression changed. In order to select features within-group, a new method named sparse-group lasso has been previously developed [35]. In future studies, we will include sparse-group lasso into GDP regularization and try different types of group prior knowledge (such as pathways), which might further improve its applicability in cancer survival analysis. Through following the recommendations using the TRIPOD (Transparent Reporting of a multivariable prediction model for Individual Prognosis Or Diagnosis) Statement [36], we will also improve the generality of GDP in the future.

Currently, the multi-omics diagnosis for cancer patients is still at its early stage and has not been widely used in clinical practice, which will limit GDP’s immediate application in real-world scenarios. However, with the advent of multi-omics technologies based on high-throughput sequencing, such as whole genome DNA sequencing, and whole transcriptome and epigenome profiling, GDP might be helpful for clinical diagnosis and genomics-guided prognosis in the near future.

## Figures and Tables

**Table 1:** Summary of the selected TCGA datasets. Only the TCGA tumor types with a ratio of non-censored over censored of no less than 0.3 and the number of patients no less than 100 were considered in the GDP analysis.

Tumor Name	Tumor Full Name	# of Patients	# Censored
GBM	Glioblastoma multiforme	579	101
OV	Ovarian serous cystadenocarcinoma	571	232
KIRC	Kidney renal clear cell carcinoma	532	355
HNSC	Head and neck squamous cell carcinoma	528	304
LUAD	Lung adenocarcinoma	507	322
LUSC	Lung squamous cell carcinoma	504	284
SKCM	Skin cutaneous melanoma	469	249
STAD	Stomach adenocarcinoma	443	270
BLCA	Bladder urothelial carcinoma	409	229
LIHC	Liver hepatocellular carcinoma	377	245
SARC	Sarcoma	261	162
LAML	Acute myeloid leukemia	198	66
PAAD	Pancreatic adenocarcinoma	185	85
ESCA	Esophageal carcinoma	185	108

## Supplementary Materials

The following are available online at https://www.mdpi.com/2073-4425/10/3/240/s1, Table S1: TCGA sample information and GDP methods comparison, Table S2: Simulation details, Table S3: Genes selected by GDP for GBM cancer prognosis.

## Abbreviations

Lasso	Least absolute shrinkage and selection operator
TCGA	The Cancer Genome Atlas
C-index	Concordance index
CPH	Cox proportional hazard model
NSS	Non-linear survival simulation
LSS	Linear survival simulation
CNV	Copy number variation
GDP	Group lasso regularized deep learning for the survival prediction in cancer patients
TRIPOD	Transparent Reporting of a multivariable prediction model for Individual Prognosis Or Diagnosis
iCAGES	Integrated CAncer Genome Score
GISTIC	Genomic Identification of Significant Targets in Cancer
GDAC	Genome Data Analysis Center
