# Supplementary material for: Group Lasso Regularized Deep Learning for Cancer Prognosis from Multi-Omics and Clinical Features
Source: Genes (Basel). 2019 Mar 21;10(3):240. doi: 10.3390/genes10030240 (PMC6471789; doi:10.3390/genes10030240)
Supplement: Supplementary file 1 [file genes-10-00240-s001.pdf]

Supplementary Materials

Table S1: Comparison of Group Lasso (GL) to Lasso (L) and no regularization (NA) in selected TCGA tumors. One-sided t test (alternative hypothesis is c-index based on GL is larger than others) was applied to the c-index based on the testing datasets in different tumor types. “Group lasso” performs better than “lasso” and “no regularization” in BLCA, GBM and KIRC tumor types, but does not have significant advantage for other tumor types.

Tumor type	P value (GL VS L)	P value (GL VS NA)
BLCA	0.020891546	0.002262145
GBM	0.029267454	0.001579158
KIRC	0.038955603	0.01449779
SKCM	0.057126955	0.036834369
ESCA	0.265537809	0.000885667
HNSC	0.358980698	0.161170726
LAML	0.361479676	0.100064324
STAD	0.538972247	0.624022872
OV	0.575967512	0.16755083
SARC	0.675777968	0.644040525
LUAD	0.690452057	0.085165098
LIHC	0.744399143	0.602212379
PAAD	0.942557494	0.114694866
LUSC	0.998014391	0.282344125

Table S2: Detail of the data simulation.

simulation	group size	# of groups	# of relevant groups	total # of relevant features	total # of features	sample size	feature matrix transformation
s1	4	2000	5	20	8000	500	no
s2A	1	8000	16	16	8000	500	no
s2B	2	4000	8	16	8000	500	no
s2C	4	2000	4	16	8000	500	no
s2D	8	1000	2	16	8000	500	no
s2E	16	500	1	16	8000	500	no
s3A	4	2000	5	20	8000	500	no
s3B	4	2000	5	20	8000	500	quadratic
s3C	4	2000	5	20	8000	500	absolute

Table S3: Top 10 genes selected by GDP for GBM

Official Symbol	Official Full Name
ING1	inhibitor of growth family member 1
RAD51	RAD51 recombinase
KLF15	Kruppel like factor 15
CDHR4	cadherin related family member 4
RRP8	ribosomal RNA processing 8
PCYT1A	phosphate cytidylyltransferase 1, choline, alpha
GRTP1	growth hormone regulated TBC protein 1
BCL2	BCL2 apoptosis regulator
EHD4	EH domain containing 4
TYRO3	TYRO3 protein tyrosine kinase
